# Caveolin-1 Regulates Perivascular Aquaporin-4 Expression After Cerebral Ischemia

**DOI:** 10.3389/fcell.2020.00371

**Published:** 2020-05-25

**Authors:** Irina Filchenko, Camille Blochet, Lara Buscemi, Melanie Price, Jerome Badaut, Lorenz Hirt

**Affiliations:** ^1^Service of Neurology, Department of Clinical Neurosciences, CHUV, Lausanne, Switzerland; ^2^North-Western State Medical University named after I.I. Mechnikov, Saint-Petersburg, Russia; ^3^Department of Fundamental Neurosciences, University of Lausanne, Lausanne, Switzerland; ^4^Brain Molecular Imaging Lab, CNRS UMR 5287, INCIA, University of Bordeaux, Bordeaux, France; ^5^Basic Science Department, Loma Linda University School of Medicine, Loma Linda, CA, United States

**Keywords:** stroke, aquaporin (AQP)-4, caveolin-1 (CAV1), recovery, astrocyctes, endfeet

## Abstract

Edema is a hallmark of many brain disorders including stroke. During vasogenic edema, blood-brain barrier (BBB) permeability increases, contributing to the entry of plasma proteins followed by water. Caveolae and caveolin-1 (Cav-1) are involved in these BBB permeability changes. The expression of the aquaporin-4 (AQP4) water channel relates to brain swelling, however, its regulation is poorly understood. Here we tested whether Cav-1 regulates AQP4 expression in the perivascular region after brain ischemia in mice. We showed that Cav-1 knockout mice had enhanced hemispheric swelling and decreased perivascular AQP4 expression in perilesional and contralateral cortical regions compared to wild-type. Glial fibrillary acidic protein-positive astrocytes displayed less branching and ramification in Cav-1 knockout mice compared to wild-type animals. There was a positive correlation between the area of perivascular AQP4-immunolabelling and branch length of Glial fibrillary acidic protein-positive astrocytes in wild-type mice, not seen in Cav-1 knockout mice. In summary, we show for the first time that loss of Cav-1 results in decreased AQP4 expression and impaired perivascular AQP4 covering after cerebral ischemia associated with altered reactive astrocyte morphology and enhanced brain swelling. Therapeutic approaches targeting Cav-1 may provide new opportunities for improving stroke outcome.

**Significance Statement:**

Severe brain edema worsens outcome in stroke patients. Available treatments for stroke-related edema are not efficient and molecular and cellular mechanisms are poorly understood. Cellular water channels, aquaporins (AQPs), are mainly expressed in astrocytes in the brain and play a key role in water movements and cerebral edema, while endothelial caveolins have been suggested to play a role in vasogenic edema. Here we used an integrative approach to study possible interaction between AQP4 and caveolin-1 (Cav-1) after stroke. Absence of Cav-1 was associated with perivascular changes in AQP4 expression and enhanced brain swelling at 3 days after cerebral ischemia. The present work indicates a direct or indirect effect of Cav-1 on perivascular AQP4, which may lead to novel edema therapy.

## Introduction

Cerebral edema is a hallmark of many brain diseases including stroke. It is characterized by a net increase in water in the brain tissue, triggering tissue swelling. Brain edema has a simple definition, hiding a very complex phenomenon with heterogeneous processes depending on the brain disease (Michinaga and Koyama, 2015; Jha et al., 2019). Underlying molecular and cellular mechanisms of edema formation and resolution are not well understood. Recently, brain edema was divided into three categories, cytotoxic, ionic and vasogenic, based on changes in BBB properties (Simard et al., 2007; Michinaga and Koyama, 2015; Clement et al., 2020). Brain endothelium, critical for the BBB function, restricts the passage of molecules from blood to the brain tissue via the presence of tight junctions between endothelial cells and specific transporters (Villabona-Rueda et al., 2019) and a low level of transcytosis (Ayloo and Gu, 2019). The increase in BBB permeability after stroke (Sadeghian et al., 2018) has been linked to an early increase in transcytosis (Knowland et al., 2014), which is caveolae and Cav-1 dependent (Knowland et al., 2014; Sadeghian et al., 2018). Cav-1 belongs to the caveolin family and contributes to the formation of caveolae (plasma membrane invaginations), transcytosis and signal transduction (Parton and del Pozo, 2013). In brain Cav-1 is found in endothelial cells (Knowland et al., 2014; Badaut et al., 2015; Blochet et al., 2020), astrocytes (Badaut et al., 2015; Blochet et al., 2020) and neurons (Xu et al., 2015) of the neurovascular unit (NVU). We recently demonstrated in Cav-1 knockout (KO) mice (JAX stock #007083) that presence of Cav-1 is associated with better survival and recovery after stroke, suggesting a protective role for endogenous Cav-1. We also showed that Cav-1 plays a role in neovascularization and astrogliosis after ischemic injury (Blochet et al., 2020). Choi and colleagues showed that Cav-1 overexpression attenuated brain edema after cerebral ischemia in the rat (Choi et al., 2016).

Several AQPs are present in the brain, the most abundant being the water channel AQP4. AQP4 is important for water movement and edema in the NVU (Badaut et al., 2014) and is expressed in astrocytes (Bi et al., 2017; Hirt et al., 2018a). An early increase in AQP4 expression occurs 1 h after stroke onset on perivascular end-feet and a late increase on astrocyte processes 48 h after stroke onset (de Castro Ribeiro et al., 2006; Hirt et al., 2009). AQP4 may have a dual role in edema, contributing to edema formation (Manley et al., 2000) and facilitating water clearance (Hirt et al., 2009). Moreover, the presence of AQP4 on perivascular astrocyte end-feet is important for perivascular cerebro-spinal fluid flow and protein clearance (Iliff et al., 2012). In perivascular end-feet expression depends on syntrophin, dystrophin–dystroglycan and agrin complex (Noell et al., 2011; Vella et al., 2015). Changes in pericyte properties and platelet-derived growth factor subunit B signaling have been linked to decreased AQP4 polarization (Lindblom et al., 2003; Gundersen et al., 2014; Munk et al., 2019). However, little is known about the molecular mechanisms involved in regulation of presence of AQP4 in perivascular end-feet after brain ischemia.

Outside the brain, endothelial Cav-1 has been shown to contribute to the presence of AQPs in the cell membrane (Jablonski and Hughes, 2006; Aoki et al., 2012; Li et al., 2012; Kim et al., 2013; Jung et al., 2015). Interestingly, Bi et al. (2017) proposed Cav-1 phosphorylation affects AQP4 subcellular distribution. Here, we investigated if Cav-1 is involved in AQP4 expression and cellular distribution after brain ischemia relating to astrogliosis and brain swelling.

## Materials and Methods

Animal experiments and care complied with the guidelines of the Swiss Veterinary Office and were approved by the Animal Care and Use Committee (license VD2017.5). Animal reporting was according to ARRIVE guidelines.

### Animal Groups and Experimental Design

Male C57Bl/6J wild-type (WT) mice (6 weeks) were from Charles River (*n* = 16). Cav-1 KO mice in a C57Bl/6J background from Jackson Laboratory (JAX stock #007083) were bred on site (*n* = 18). Animals were housed for at least 1 week in a temperature-controlled animal facility on a 12-h light-dark cycle with *ad libitum* access to food and water. Cages contained standard bedding and enrichment material.

(1) Immunofluorescence experiments with *n* = 9 WT and n = 9 Cav-1 KO animals, two to three different samples at three different time points (sham and middle cerebral artery occlusion (MCAO) at 6 and 72 h post injury). Animals were perfused (see below) at 6 and 72 h. Our veterinary authority requested the following humane termination endpoints: loss of righting reflex from 24 h post-injury, status epilepticus, body weight loss of more than 25%.
(2) Brain swelling was assessed in mice from a previous experiment (Blochet et al., 2020) with *n* = 7 WT and *n* = 9 Cav-1 KO mice. Briefly, brains were frozen in liquid nitrogen vapor and twelve 20 μm-thick coronal cryostat (Leica CM3050) sections per brain collected on Superfrost-Plus slides (Fisher Scientific). Sections were stained with cresyl violet, imaged with a stereomicroscope (Nikon SMZ 25) at 5× magnification with blinded analysis using ImageJ/FIJI software.

### Experimental Model of Cerebral Ischemia

Focal cerebral ischemia was induced by left MCAO with a monofilament (Doccol Corporation) for 35 min under isoflurane anesthesia as described (Blochet et al., 2020). Ischemia was considered successful if cerebral blood flow during MCAO was below 20% of baseline and over 50% of baseline at reperfusion according to laser Doppler flowmetry (Perimed). Rectal temperature was kept at 37.0 ± 0.5°C during surgery. We performed sham surgery under anesthesia by placing the Doppler probe onto the skull and dissecting the carotid arteries without filament insertion. After surgery, animals were maintained overnight at 28°C. We assessed the coat, eyes and nose, neurological deficit, epileptic seizures, body weight loss and dehydration of all animals daily.

### Immunofluorescence Staining

For tissue collection for immunofluorescence staining mice were transcardially perfused with PBS followed by 4% paraformaldehyde (PFA) at 4°C, 10 mL/min, at 6 and 72 h post injury. Following overnight post-fixation in PFA, brains were incubated in phosphate buffered saline (PBS) with 30% sucrose for 48 h, then frozen in isopentane on dry ice. Coronal cryostat sections (25 μm-thick) were collected, stored in PBS with 30% sucrose at −20°C.

Immunofluorescence staining was performed on above sections with antigen retrieval using cold 33% acetic acid, 66% ethanol and blocking with 1% bovine serum albumin, 5% horse serum, 0.1% Triton X100 solution for 1 h at room temperature. Primary antibodies were: Mouse anti-microtubule associated protein 2 (MAP-2, 1:500, Millipore Merck, cat # MAB3418), Rabbit anti-AQP4 (1:500, Merck cat # AB3594-200UL), Rat anti-cluster of differentiation 31 (CD31) (1:100, BD Biosciences, cat # 550274), Mouse anti-Glial Acidic Fibrillary Protein (GFAP) (1:2000, Merck, cat # MAB3402), Mouse anti-glutamine synthetase (GS, 1:1000, Merck, cat # MAB302). Primary antibodies were incubated in 1% bovine serum albumin (BSA), 0.3% Triton X100 in PBS overnight at 4°C. Alexa Fluor-labeled (Invitrogen) secondary antibodies were incubated in the same solution with 4’,6-diamidino-2-phenylindole (DAPI) for 1 h at room temperature. Sections were mounted on SuperFrost-Plus slides (Fischer Scientific) with FluorSave medium (Calbiochem) and coverslipped.

### Image Acquisition and Analysis

We captured images of coronal brain sections using a slide-scanner (Zeiss AxioScan Z1) at 10× magnification. We acquired higher magnification images (63×) of ipsilateral and contralateral striatum and cortex to the lesion with a confocal microscope (Zeiss LSM 710 Quasar).

For AQP4 expression patterns, vessel density analysis and astrocyte morphological analysis Images were acquired in ipsilateral and contralateral striatum and cortex to the lesion with the same confocal microscope at 40× magnification (Figures 2, 3). We analyzed AQP4 expression and then vessel density using the Fiji vessel density plugin (open source image processing package). This software analyzes vessel-like signals and calculates vascular density = vessel area/total area × 100% and vascular length density = skeletonized vessel area/total area × 100%. Quantification was done on averaged z-projections using Fiji on stacks of seven to nine images with 2 μm-spacing with three images for each region of interest per animal (ipsilateral and contralateral striatum and cortex in animals following stroke and striatum and cortex in sham animals, Supplementary Figure S1), three animals per group (Cav-1 KO and WT). Astrocyte morphology was analyzed in the same regions using Fiji’s bandpass and unsharp mask filtering, binarization, skeletonization and analysis on the averaged z-projections as described previously (Blochet et al., 2020). Quantification was done on averaged z-projections using Fiji on stacks of seven to nine images with 2 μm-spacing on three images for each brain region per animal (ipsilateral and contralateral cortex in animals following stroke and cortex in sham animals), three animals per group (Cav-1 KO and WT). AQP4 expression pattern, vessel density and astrocyte morphology analyses were performed blinded.

### Statistical Analysis

Data were expressed as median, interquartile range, minimal and maximal values and analyzed with IBM SPSS Statistics 25 and Graphpad Prism 8. Data normalization was conducted in a two-step approach as described previously. We carried out one-way ANOVA with Tukey’s correction for multiple comparisons of six groups (Cav-1 KO/WT ipsilateral/contralateral and Cav-1 KO/WT sham cortex/striatum) for immunofluorescence analysis of AQP4 and CD31 expression patterns in the cortex and comparison of six groups (Cav-1 KO/WT ipsilateral/contralateral cortex and Cav-1 KO/WT sham cortex) for immunofluorescence analysis of astrocyte morphology. The association between AQP4 expression patterns and astrocyte morphology was assessed with Pearson correlation coefficient. Statistical significance was set at ^∗^*p* < 0.05, ^∗∗^*p* < 0.01, ^∗∗∗^*p* < 0.005 and ^****^p < 0.001.

## Results

### AQP4 Expression in WT and in Cav-1 KO Mice and Brain Swelling

**FIGURE 1:**
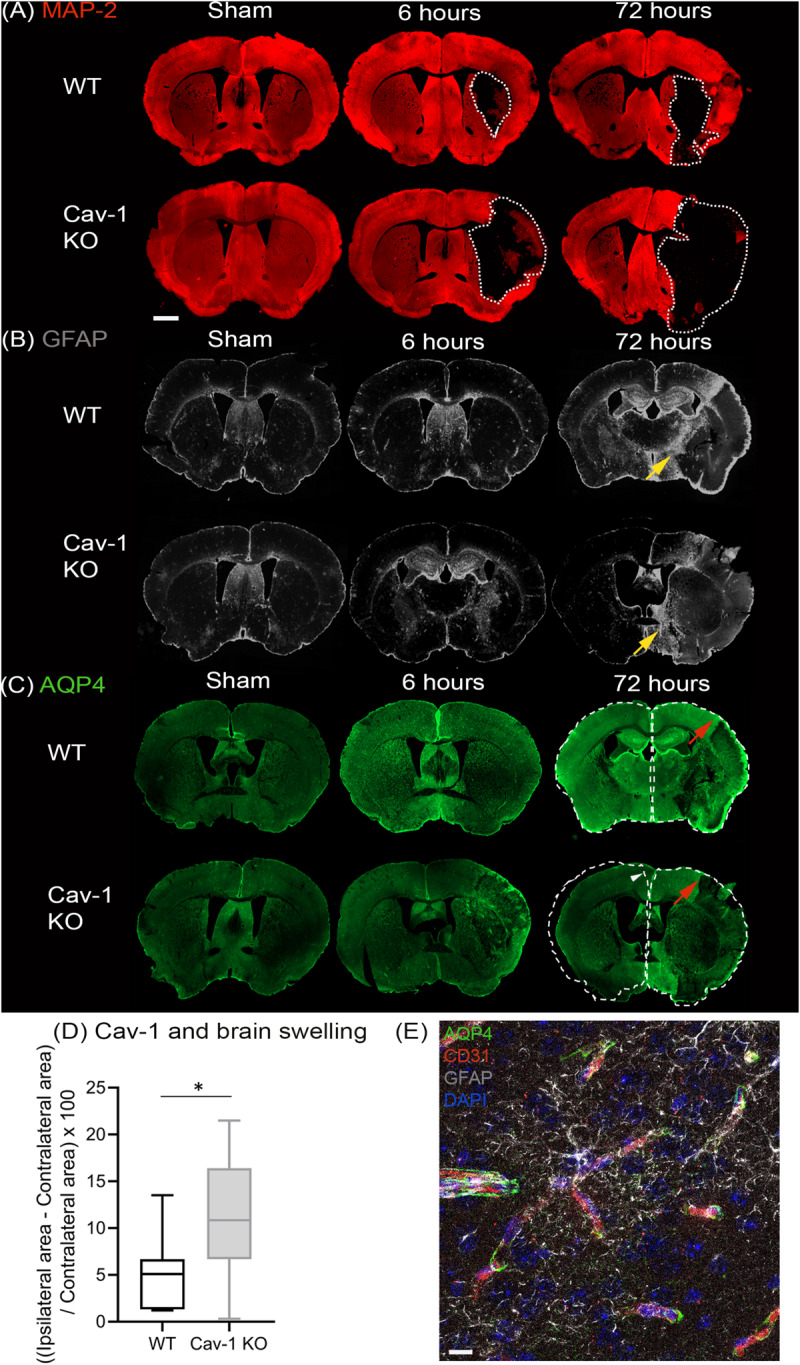
Immunostaining of coronal slices of WT and Cav-1 KO mice after sham surgery and at 6 and 72 h after MCAO: **(A)** MAP-2 expression (red) showing the lesion outlined by a white dotted line. **(B)** GFAP expression (gray) showing the extent of the astrocyte scar highlighted by yellow arrows. **(C)** AQP4 expression (green); the contralateral hemisphere area is delineated by a white dotted line relative to the ipsilateral hemisphere to appreciate the swelling and the white arrowhead points to the shift of the midline due to swelling. Red arrows highlight AQP4 staining in the cortical perilesion. Scale bar = 1 mm. **(D)** Brain swelling at 7 days after MCAO in WT and Cav-1 KO mice. **(E)** Immuno-staining at ×63 magnification illustrating the relationship between AQP4 (green), vessels labeled by CD31 (red) and reactive astrocytes (gray), scale bar = 10 μm.

Using an anti-body to the neuronal marker microtubule associated protein 2 (MAP-2), we delineated the lesion area by lack of MAP-2 labeling on coronal brain sections (white dashed lines, Figure 1A). GFAP staining was performed at 6 and 72 h post injury (Figure 1B). Decreased GFAP staining was observed in the ipsilateral striatum of both genotypes after stroke with a larger decrease in Cav-1 KO mice than WT mice at 72 h after stroke onset (Figure 1B). The area of enhanced GFAP labeling corresponding to the glial scar in the perilesion was smaller in Cav-1 KOs than WTs (yellow arrows, Figure 1B).

AQP4 staining was observed in the periventricular areas and in the glia limitans with no significant difference between the two genotypes (Figure 1C). AQP4-labeling was absent in the striatum of Cav-1 KO mice at six but not in WTs, indicating a difference in AQP4 expression after stroke. Similarly, AQP4 labeling in the perilesion and contralateral cortex was decreased in Cav-1 KO mice compared to WT at 6 and 72h after stroke onset (red arrows, Figure 1C).

Brain swelling (Figure 1D) was higher in Cav-1 KO than in WT mice (outlined by white dotted lines corresponding to the ipsilateral hemisphere relative to the contralateral hemisphere in Figure 1C). Quantification of GFAP and AQP4 staining was done on higher magnification images to assess cell morphology (GFAP) and cellular location (AQP4) respectively.

### Perivascular AQP4 Expression

**FIGURE 2:**
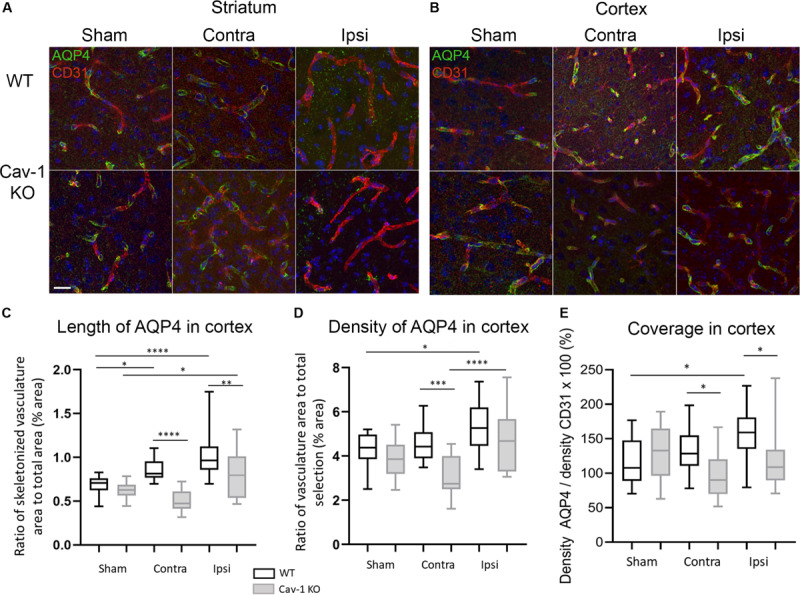
**(A)** High magnification images of AQP4 on vessels stained by CD31 at 72 h post-MCAO (×63 magnification) in the striatum. **(B)** High magnification images of AQP4 on vessels stained with CD31 at 72 h post-MCAO (×63 magnification) in the cortex. **(C)** Analysis of the length of AQP4-immunolabeled vessel-like structures with Fiji vascular density plug-in in WT and Cav-1 KO after MCAO and in sham animals; *n* = 3 areas per animal with three animals per group. Black and gray plots refer to WT and Cav-1 KO animals, respectively. Significant differences between WT and Cav-1 KO animals are noted as *p < 0.05, **p < 0.01, ***p < 0.005 and ****p < 0.001. WT-Sham compared to WT-Ipsi: 95% CI [−0.4949 to −0.1666], *p* < 0.0001, WT-Sham compared to WT-Contra: 95% CI [−0.3330 to −0.004702], *p* = 0.04, KO-Sham compared to KO-Ipsi: 95% CI [−0.3355 to −0.007202], *p* = 0.0354, WT-Contra compared to KO-Contra: 95% CI [0.1888 to 0.5171], *p* < 0.0001, and WT-Ipsi compared to KO-Ipsi: 95% CI [0.05398 to 0.3822], *p* = 0.0027. **(D)** Analysis of the density of AQP4-immunolabeled vessel-like structures assessed by the same Fiji plug-in. WT-Sham compared to WT-Ipsi: 95% CI [−1.985 to −0.09168], *p* = 0.0229, WT-Contra compared to KO-Contra: 95% CI [0.5432 to 2.436], *p* = 0.0002, KO-Contra compared to KO-Ipsi: 95% CI [−2.594 to −0.7009], *p* < 0.0001. **(E)** Analysis of the coverage of AQP4 on vessels measured as the ratio between the density of AQP-4 vessel-like pattern and CD31-labeled vessels. WT-Sham compared to WT-Ipsi: 95% CI [−73.27 to −1.223], *p* = 0.0384, WT-Contra compared to KO-Contra: 95% CI [3.157 to 75.20], *p* = 0.0247, WT-Ipsi compared to KO-Ipsi: 95% CI [3.509 to 75.55], *p* = 0.0228. Contra: contralateral hemisphere to the lesion, Ipsi: ipsilateral hemisphere to the lesion. Scale bar = 20 μm. Comparisons were carried out by one-way ANOVA with Tukey’s multiple comparisons post-test.

AQP4 was present on GFAP-positive astrocyte end-feet encircling the CD31-immunolabelled endothelial cells (Figure 1E) in all brain regions investigated. In contrast, AQP4 staining pattern in the lesion (ipsilateral striatum) was punctate without perivascular staining in WT and KOs (Figure 2A). Note, in the lesion the decrease in AQP4-staining is more pronounced in the Cav-1 KO mice.

AQP4 staining was observed around the blood vessels in the perilesion and contralateral cortex in both genotypes. A significant increase in the length of perivascular-AQP4 staining was observed in the ipsilateral and contralateral cortex of WT stroke animals compared to WT sham (Tukey’s multiple comparisons test, 95% CI [−0.4949 to −0.1666], *p* < 0.0001 and 95% CI −0.3330 to −0.004702], *p* = 0.04 respectively) (Figure 2B). The same was observed in sham Cav-1 KO mice compared to ipsilateral cortex (KO-Sham compared to KO-Ipsi: 95% CI [−0.3355 to −0.007202], *p* = 0.0354) but not for the contralateral cortex. Interestingly, the length of AQP4 staining was significantly decreased in the ipsi- and contralateral cortex of the Cav-1 KO mice compared to WTs after stroke (WT-Contra compared to KO-Contra: 95% CI [0.1888 to 0.5171], *p* < 0.0001, and WT-Ipsi compared to KO-Ipsi: 95% CI [0.05398 to 0.3822], *p* = 0.0027) (Figure 2C). Blood vessels were stained with CD31 (Figure 2A) and measurements (not shown) showed a significant increase in vascular density in the lesion compared to sham, consistent with what has been previously reported (Blochet et al., 2020). The coverage of CD31-positive blood vessels by perivascular AQP4 was significantly increased in the perilesion/ipsilateral cortex WT stroke animals compared to sham (WT-Sham compared to WT-Ipsi: 95% CI [−73.27 to −1.223], *p* = 0.0384) (Figure 2E). However, perivascular AQP4 coverage was significantly lower in Cav-1 KO mice compared to WT in the ipsi- and contralateral cortex (WT-Contra compared to KO-Contra: 95% CI [3.157 to 75.20], *p* = 0.0247, WT-Ipsi compared to KO-Ipsi: 95% CI [3.509 to 75.55], *p* = 0.0228.) (Figure 2E).

### Morphological Alterations of GFAP- Positive Astrocytes

**FIGURE 3:**
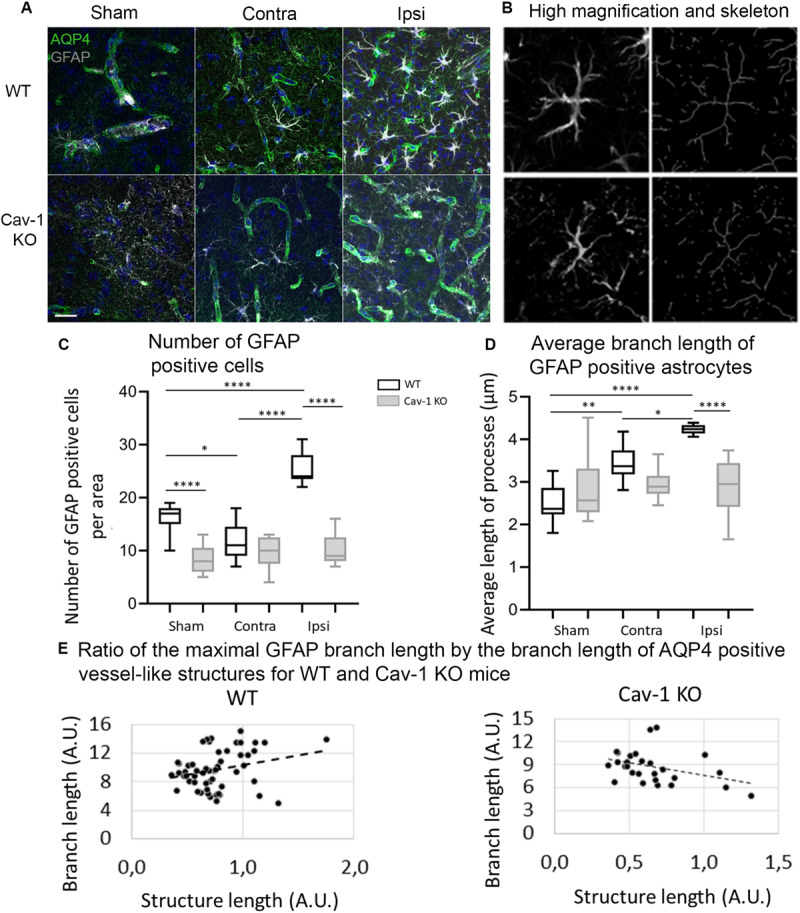
**(A)** Immunofluorescence staining with AQP4 (green) and GFAP (gray) in WT and Cav-1 KO mice at 72 h post-MCAO (63× magnification). AQP4 co-localized with GFAP-positive astrocyte end-feet. Scale bar = 20 μm. **(B)** Single-channel confocal microscopy ROIs obtained from 40× magnification images, illustrating the overview of a GFAP-positive astrocyte morphology and its skeletonization. **(C)** Number of GFAP-positive reactive astrocytes. WT-Sham compared to WT-Ipsi: 95% CI: [−13.42 to −5.027], *p* < 0.0001, WT-Sham compared to WT-Contra: 95% CI [0.4716 to 8.862], *p* = 0.0212, WT-Sham compared to KO-Sham: 95% CI: [3.805 to 12.20], *p* < 0.0001, WT-Ipsi compared to WT-Contra: 95% CI [−18.08 to −9.694], *p* < 0.0001, and WT-Ipsi compared to KO-Ipsi: 95% CI [10.92 to 19.31], *p* < 0.0001. **(D)** Average branch length of GFAP-positive astrocytes. WT-Sham compared to WT-Ipsi: 95% CI [−2.455 to −1.005], *p* < 0.0001, WT-Sham compared to WT-Contra: 95% CI [−1.648 to −0.1988], *p* = 0.0054, WT-Ipsi compared to WT-Contra: 95% CI: [−1.531 to −0.08208], *p* = 0.0210, and WT-Ipsi compared to KO-Ipsi: 95% CI [0.5799 to 2.029], *p* < 0.0001. Comparisons were carried out by one-way ANOVA with Tukey’s multiple comparisons post-test. **(E)** The positive correlation between the length of AQP4-immunolabeled vessel-like structures and the maximal branch length of GFAP-immunolabeled astrocytes in WT mice (Pearson correlation coefficient = 0.451, *p* = 0.024) and Cav-1 KO mice (Pearson correlation coefficient = −0.443, *p* = 0.021).

In both genotypes there was GFAP staining in the ipsi- and contralateral cortex (Figure 3A) and the labeling was significantly decreased in the lesion (Figure 1A). Images of GFAP-positive astrocytes were skeletonized for morphological analysis (Figure 3B). There were significantly fewer GFAP positive astrocytes in the ipsilateral cortex of Cav-1 KO mice compared to WT (WT-Ipsi compared to KO-Ipsi: 95% CI [10.92 to 19.31], *p* < 0.0001) (Figure 3C). Morphological analysis showed a significant increase in the branch length of astrocytes in the ipsi- and contralateral cortex of WT stroke mice compared to sham (WT-Sham compared to WT-Ipsi: 95% CI [−2.455 to −1.005], *p* < 0.0001, WT-Sham compared to WT-Contra: 95% CI [−1.648 to −0.1988], *p* = 0.0054, WT-Ipsi compared to WT-Contra: 95% CI: [−1.531 to −0.08208], *p* = 0.0210). It also showed shorter process length in Cav-1 KO stroke mice in the ipsilateral cortex compared to WT (WT-Ipsi compared to KO-Ipsi: 95% CI [0.5799 to 2.029], *p* < 0.0001) (Figure 3C). Similar results were obtained for GS-positive astrocytes (Supplementary Figure S2). As observed for AQP4-labeling, there was no change in the length of GFAP-positive branches in Cav-1 KO stroke mice compared to sham (Figure 3D). These results suggested a potential relationship between AQP4 expression and astrocyte morphology. Analysis of AQP4 staining and reactive astrocyte morphology exhibited positive correlation between the length of AQP4-immunolabelled vessel-like structures and number of GFAP-positive branches in WT mice (Pearson correlation coefficient = 0.532, *p* = 0.005; Figure 3E). Conversely, in Cav-1 KO mice a negative correlation between length of AQP4-immunolabelled vessel-like structures and branch length (Pearson correlation coefficient = −0.443, *p* = 0.021) was observed (Figure 3E). No association between AQP4 expression patterns and selected morphological features of GS-immunolabeled astrocytes was observed.

## Discussion

Cav-1 deficient mice are vulnerable to stroke as they develop larger lesions and recover less well than WT mice (Blochet et al., 2020). We show now that mice lacking Cav-1 have significantly less astrocytic AQP4 in the lesion core, perilesion and contralateral cortex after stroke. In addition, there was less perivascular coverage by AQP4 in these areas. Concomitant with the changes in AQP4 expression and coverage, reactive astrocyte morphological changes were severely diminished and brain swelling was increased in the Cav-1 KO mice after stroke. The work indicates a link between Cav-1, reactive astrocyte morphology and perivascular astrocytic AQP4 expression, brain edema and consequent recovery after stroke.

### Diminished Perivascular AQP4 Expression: Consequence on Brain Edema After Stroke

There is abundant literature on the role of AQP4 in brain edema (for review Badaut et al., 2014). The contribution of AQP4 varies greatly depending on time post-injury, brain region, amongst others leading to apparently contradictory results on edema, lesion size and outcome in AQP4 KO mice after stroke (Manley et al., 2000; Yao et al., 2008; Zeng et al., 2012; Hirt et al., 2017). AQP4 expression was rapidly up-regulated in the lesion and perilesion at 6 h in WT mice in agreement with our earlier description of an increase 1 h after stroke onset in the infarct core and ischemic penumbra (de Castro Ribeiro et al., 2006; Hirt et al., 2009). However, there was less increase in AQP4-staining in Cav-1 KOs indicating that the absence of Cav-1 affects AQP4 expression. The lower AQP4 expression is associated with a larger edema formation, which is in agreement with our earlier observation that up-regulating AQP4 on astrocytic end-feet, attenuated the early phase of hemispheric enlargement 1 h after MCAO (Hirt et al., 2009). WT mice exhibited higher perivascular expression of AQP4 (Figure 2). Interestingly, disrupted perivascular AQP4 polarization caused by reactive astrogliosis was shown to impair glymphatic clearance in the models of Alzheimer disease and senescence (Kress et al., 2014). Altogether, increased perivascular AQP4 after stroke could be a protective mechanism by promoting edema resolution and facilitating water and debris clearance from the perivascular space.

### Role of Cav-1 in Perivascular AQP4 Changes: Potential Mechanisms

Lower perivascular AQP4 expression was observed after stroke in absence of Cav-1, suggesting a role for Cav-1 in AQP4 expression in astrocyte perivascular end-feet (Figure 2). In support of our observations, Cav-1 has recently been implicated in movement of AQP4 to the cell surface after oxidative stress (Bi et al., 2017). Insertion of AQP4-heterotetramers depend on the syntrophin, dystrophin-dystroglycan complex in association with the extracellular matrix protein agrin (Noell et al., 2011; Vella et al., 2015; Tham et al., 2016). Interestingly, Cav-1 binds β-dystroglycan in smooth muscle cells, linking the extracellular matrix and actin cytoskeleton (Sharma et al., 2010). Neuronal nitric-oxide synthase has been shown to down-regulate the level of expression α-syntrophin (Brenman et al., 1996; Hashida-Okumura et al., 1999; Sato et al., 2004; Zhang, 2017). As a hypothesis, Cav-1 might act on α-syntrophin levels via inhibition of eNOS, which in turn might influence the level of AQP4 on the perivascular end-feet membranes.

Another possibility for the decrease in perivascular AQP4 is a change in laminin expression in astrocytes and endothelial cells. The lack of astrocytic laminin expression in conditional knockout mice has been shown to prevent pericyte differentiation and inhibit AQP4 expression, causing BBB breakdown (Yao et al., 2014). Cav1-KO mice showed less laminin-labeled endothelial cells in the ipsilateral hemisphere following ischemic stroke than WT mice (Jasmin et al., 2007). Therefore, lower perivascular AQP4 after stroke in cav-1 KO mice could be a consequence of decreased laminin.

### Altered Reactive Astrocyte Morphology in Cav-1 KO Mice: Relation With AQP4

In contrast to our previous analysis in the striatal perilesion (Blochet et al., 2020), we observed a lower number of GFAP-labeled astrocytes in the ipsilateral cortex of MCAO Cav-1 KO animals compared to WT animals. Cav-1 KO mice also had a lower degree of GFAP branching after stroke in the perilesional and contralateral cortex than WTs (Figure 3) in agreement with our earlier work (Blochet et al., 2020). Interestingly, the number of GFAP-positive processes correlated positively with perivascular AQP4 expression in WT animals after stroke but negatively in Cav-1 KO mice. This suggests that the attenuated morphological alterations of astrocytes after stroke in Cav-1 KO mice are linked to decreased AQP4 expression. Previously, reduced AQP4 expression and loss of perivascular AQP4 localization were related to disorganized intermediate filaments in reactive astrocytes (de Castro Ribeiro et al., 2006; Allnoch et al., 2019). Cav-1 may influence AQP4 expression levels by inhibiting nitric oxide synthase, as nitric oxide was described to increase AQP4 expression and to influence astrocyte volume (Yu et al., 2012; Oku et al., 2015). AQPs seem to contribute to reactive astrocyte morphology and we showed the importance of AQP9 in astrocyte morphological changes *in vitro* and *in vivo* (Hirt et al., 2018b).

**FIGURE 4:**
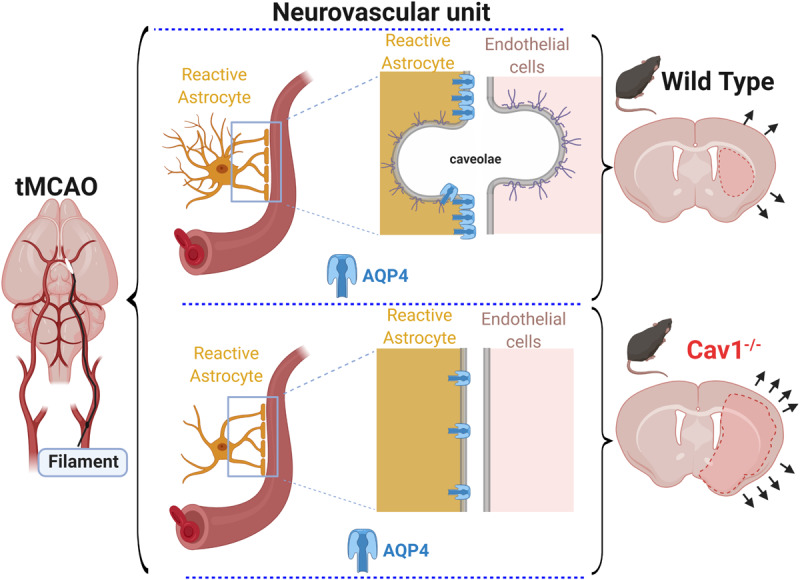
After transient filament MCAO (left), Cav 1 KO mice had larger lesions, enhanced cerebral edema and higher hemispheric swelling (bottom right) compared to WT mice (top right). At the level of the NVU (center images), the absence of caveolae* and Cav-1 correlated with altered astroglial reaction and expression of AQP4 on astrocytic end-feet in Cav-1 KO mice (bottom center) compared to WT mice (top center).

In summary, Cav-1 appears to influence AQP4 expression and distribution along vessels contributing to changes in reactive astrogliosis and brain edema observed in Cav-1 KO mice after experimental stroke (Figure 4). We are currently investigating a therapeutic approach targeting Cav-1 and have shown that the administration of cavtratin, a peptide containing the scaffolding domain of Cav-1 after MCAO improves outcome and enhances angiogenesis (Blochet et al., 2019; Blochet et al., in preparation), perhaps via some of the effects seen in this work. Indeed, understanding Cav-1 changes after stroke may lead to new treatment options for improving outcome after stroke.

## Data Availability Statement

All datasets presented in this study are included in the article/Supplementary Material.

## Ethics Statement

The animal study was reviewed and approved by Commission cantonal d’expérimentation animale, canton de Vaud. Autorisation N°2017.5.

## Author Contributions

IF: immunos, image analysis, data analysis, manuscript preparation. CB: surgery, immunos, image analysis, data analysis, manuscript preparation. MP and LB: scientific discussion and manuscript preparation. LH and JB: study concept and manuscript preparation.

## Conflict of Interest

The authors declare that the research was conducted in the absence of any commercial or financial relationships that could be construed as a potential conflict of interest.

## Supplementary Material

FIGURE S1 **(A)** Coronal brain sections at 72 h after sham and MCAO injury showing the different regions of interest (ROIs) for on high magnification images and respective quantifications. **(B)** Analysis of cortical astrocytic morphology on GS- and GFAP-immunolabeled images and by skeletonization; *n* = 3 areas per animal with 3 animals per group. The data are shown as median and interquartile range; only significant differences are displayed.

FIGURE S2 **(A)** Immunofluorescence staining with AQP4 (green) and Glutamine Synthetase (GS) (gray) in WT and Cav-1 KO mice at 72 h post-MCAO (×63 magnification). AQP4 co-localized with GS-positive astrocyte end-feet. Scale bar = 20 μm. **(B)** Single-channel confocal microscopy ROIs obtained from 40× magnification images, illustrating the overview of GS-positive astrocyte morphology and skeletonization. **(C)** Number of GS-positive astrocytes. WT-Ipsi compared to WT-Contra: 95% CI [−18.54 to −1.904], *p* = 0.0081, and WT-Ipsi compared to KO-Ipsi: 95% CI [6.348 to 22.99], *p* < 0.0001. **(D)** Ramification of GS-positive astrocytes assessed by the number of quadruple points of GS-positive astrocytes. WT-Sham compared to WT-Ipsi: 95% CI [−0.9914 to −0.03480], *p* = 0.0289, and WT-Ipsi compared to KO-Ipsi: 95% CI [0.09476 to 1.051], *p* = 0.0105. Comparisons were carried out by one-way ANOVA with Tukey’s multiple comparisons post-test.
