# Supplementary figures and images for: Caveolin-1 Regulates Perivascular Aquaporin-4 Expression After Cerebral Ischemia
Source: Front Cell Dev Biol. 2020 May 25;8:371. doi: 10.3389/fcell.2020.00371 (PMC7261922; doi:10.3389/fcell.2020.00371)

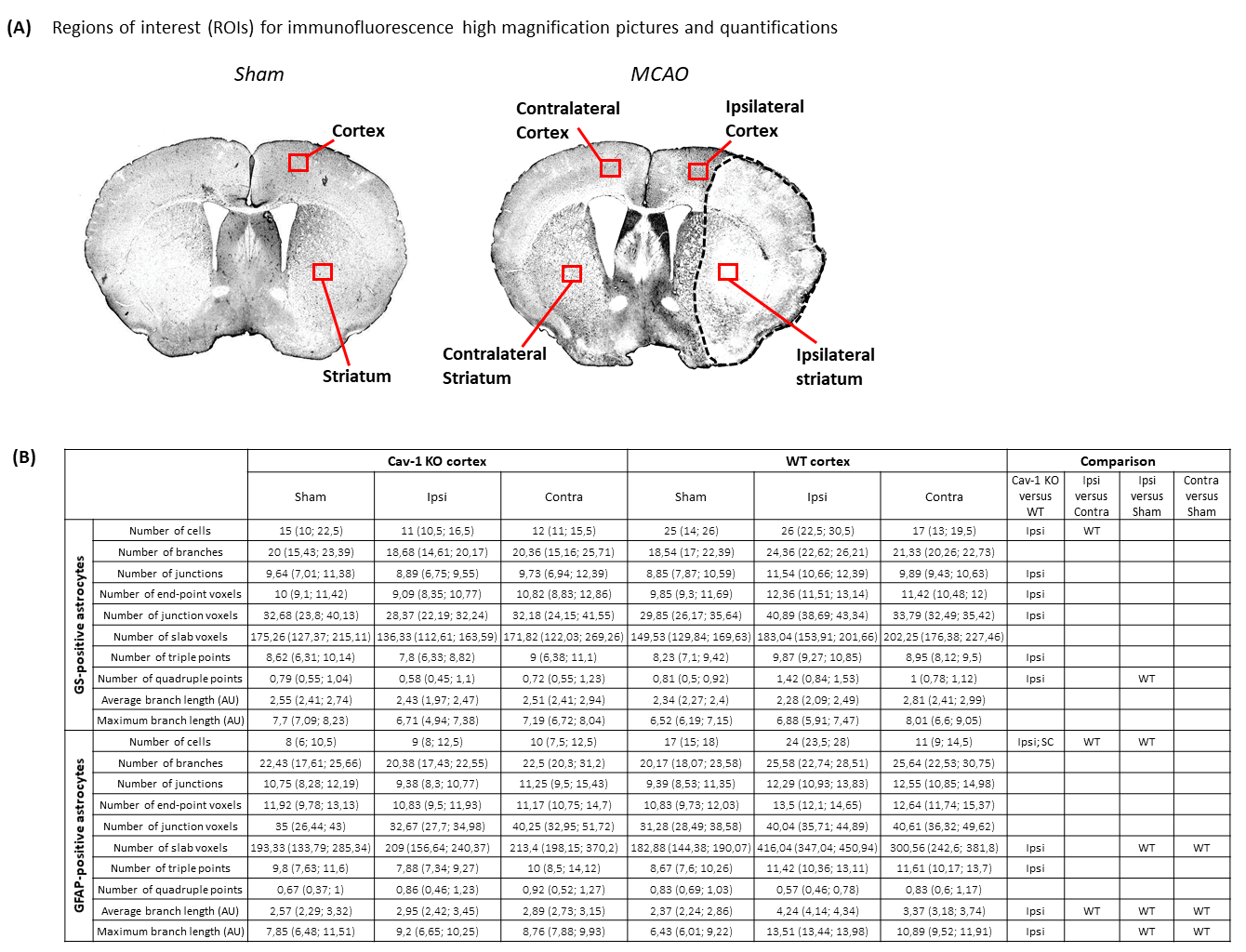

Supplement: FIGURE S1 — (A) Coronal brain sections at 72 h after sham and MCAO injury showing the different regions of interest (ROIs) for on high magnification images and respective quantifications. (B) Analysis of cortical astrocytic morphology on GS- and GFAP-immunolabeled images and by skeletonization; n = 3 areas per animal with 3 animals per group. The data are shown as median and interquartile range; only significant differences are displayed. [file Image_1.TIF]

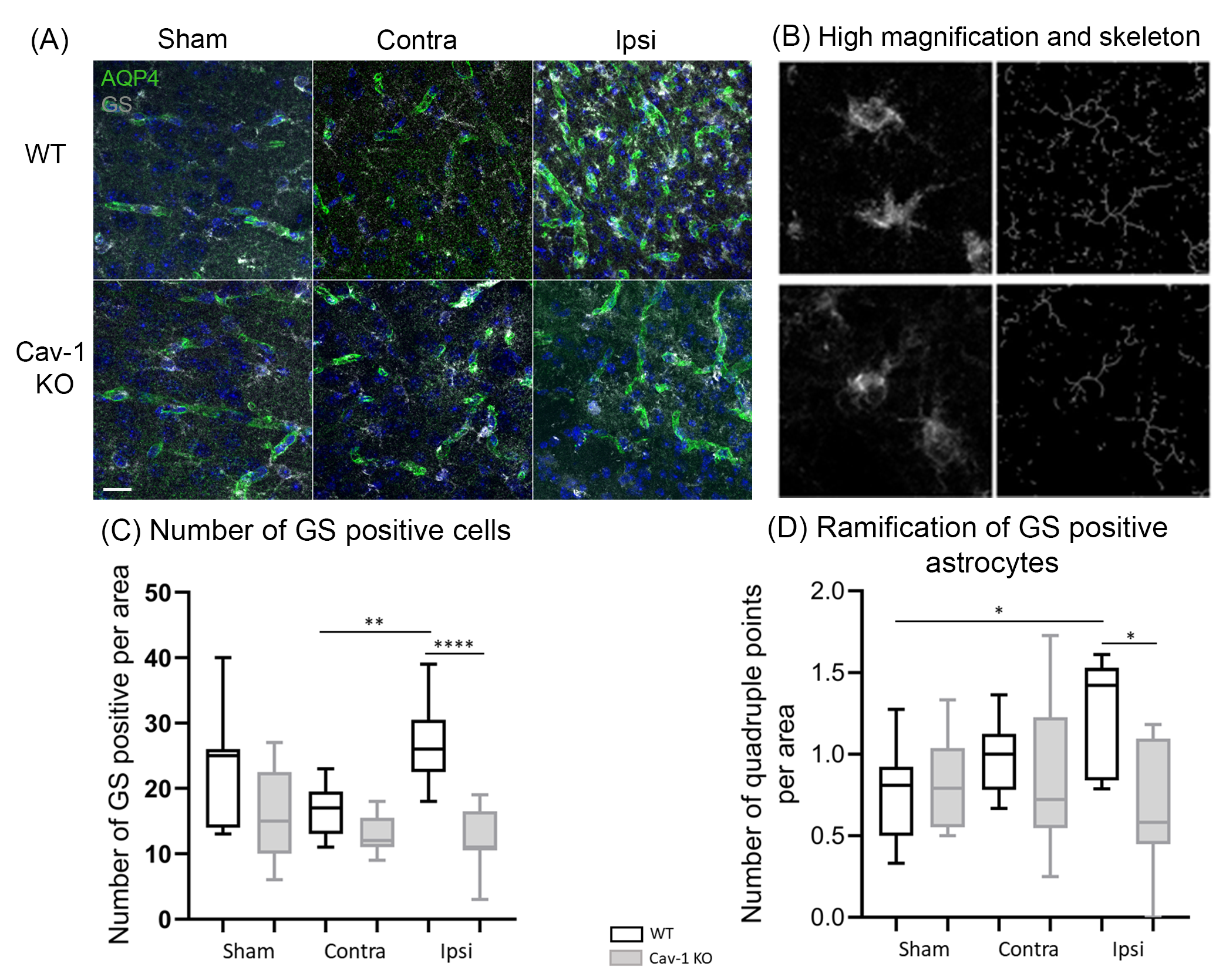

Supplement: FIGURE S2 — (A) Immunofluorescence staining with AQP4 (green) and Glutamine Synthetase (GS) (gray) in WT and Cav-1 KO mice at 72 h post-MCAO (×63 magnification). AQP4 co-localized with GS-positive astrocyte end-feet. Scale bar = 20 μm. (B) Single-channel confocal microscopy ROIs obtained from 40× magnification images, illustrating the overview of GS-positive astrocyte morphology and skeletonization. (C) Number of GS-positive astrocytes. WT-Ipsi compared to WT-Contra: 95% CI [−18.54 to −1.904], p = 0.0081, and WT-Ipsi compared to KO-Ipsi: 95% CI [6.348 to 22.99], p < 0.0001. (D) Ramification of GS-positive astrocytes assessed by the number of quadruple points of GS-positive astrocytes. WT-Sham compared to WT-Ipsi: 95% CI [−0.9914 to −0.03480], p = 0.0289, and WT-Ipsi compared to KO-Ipsi: 95% CI [0.09476 to 1.051], p = 0.0105. Comparisons were carried out by one-way ANOVA with Tukey’s multiple comparisons post-test. [file Image_2.TIF]
